# Single-cell RNA sequencing identifies critical transcription factors of tumor cell invasion induced by hypoxia microenvironment in glioblastoma

**DOI:** 10.7150/thno.81407

**Published:** 2023-06-26

**Authors:** Yanru Zhang, Bo Zhang, Chengqian Lv, Nan Zhang, Kaiyuan Xing, Zixuan Wang, Rongkai Lv, Mingchen Yu, Chaohan Xu, Yihan Wang

**Affiliations:** 1 College of Bioinformatics Science and Technology, Harbin Medical University, Harbin, 150081, China.; 2 Department of Pharmacology, State-Province Key Laboratories of Biomedicine-Pharmaceutics of China, Key Laboratory of Cardiovascular Medicine Research, Ministry of Education, College of Pharmacy, Harbin Medical University, Harbin, 150081, China.; 3 Department of Gastroenterology and Hepatology, The Second Affiliated Hospital of Harbin Medical University, Harbin, 150086, China.; 4 College of Life Science and Technology, Huazhong University of Science and Technology, China.; 5 Beijing Neurosurgical Institute, Capital Medical University. Beijing 100069, China.

**Keywords:** glioblastoma, hypoxia status, invasion, regulators, single-cell RNA sequencing

## Abstract

**Rationale:** Glioblastoma (GBM) is an aggressive malignant primary brain cancer with poor survival. Hypoxia is a hallmark of GBM, which promotes tumor cells spreading (invasion) into the healthy brain tissue.

**Methods:** To better elucidate the influence of hypoxia on GBM invasion, we proposed a data-driven modeling framework for predicting cellular hypoxia (CHPF) by integrating single cell transcriptome profiling and hypoxia gene signatures.

**Results:** We characterized the hypoxia status landscape of GBM cells and observed that hypoxic cells were only present in the tumor core. Then, by investigating the cell-cell communication between immune cells and tumor cells, we discovered significant interaction between macrophages and tumor cells in hypoxic microenvironment. Notably, we dissected the functional heterogeneity of tumor cells and identified a hypoxic subpopulation that had highly invasive potential. By constructing cell status specific gene regulatory networks, we further identified 14 critical regulators of tumor invasion induced by hypoxic microenvironment. Finally, we confirmed that knocking down two critical regulators *CEBPD* and *FOSL1* could reduce the invasive ability of GBM under hypoxic conditions. Additionally, we revealed the therapeutic effect of Axitinib and Entinostat through the mice model.

**Conclusion:** Our work revealed the critical regulators in hypoxic subpopulation with high invasive potential in GBM, which may have practical implications for clinical targeted-hypoxia cancer drug therapy.

## Introduction

Glioblastoma (GBM), a grade IV astrocytoma, is a rapidly growing and highly invasive brain tumor 1, with low median survival (range from 15 to 17 months) and high incidence (3.19 per 100,000 per year) 2-4. The extensive infiltration of the tumor surrounding parenchyma makes it impossible for GBM to be completely surgically resected, and tumor recurrence can occur even after complete resection within the visible range 5. Thus, invasion is the main obstacle to GBM treatment.

Low tumor oxygenation, also known as hypoxia, is a common feature of solid tumors 6, 7 and a key factor in tumor microenvironment that promotes cancer cell spread (invasion) into the healthy tissue to evade this adverse microenvironment 8-10. Since hypoxia is a strong inducer to stimulate an invasive ability in GBM, an increasing amount of research has been focused on how hypoxia triggers the GBM cells to invade 6, 11, 12. Several previous studies reported that hypoxia enhanced migration and invasion in GBM by activating special signaling pathways or regulators, such as* HIF1α, HIF2α*, EMT transcription factor *ZEB1* 13-16. Therefore, understanding of the hypoxia status of tumor microenvironment could help improve GBM therapy.

Previous studies on tumor hypoxia were mainly based on bulk samples. For example, Shi et al. established hypoxia-derived signatures based on microarray and RNA-seq datasets, which were promising biomarkers to predict survival and therapeutic response in stage I lung adenocarcinoma patients 17. Bhandari et al. quantified hypoxia in 8006 tumors across 19 tumor types derived from TCGA and revealed tumor hypoxia may drive aggressive molecular features across cancers 18. However, due to the dynamic degree of tissue oxygenation and the diversity of hypoxia levels across tissues, bulk sequencing is unable to reveal the high intratumor heterogeneity driven by hypoxia 6, 9, 19. Single-cell sequencing technologies have recently facilitated a deeper exploration of intratumor heterogeneity 20, 21, allowing to investigate a more aggressive phenotype under hypoxia microenvironment and infer potential regulators.

Collectively, to explore the influence of hypoxia on GBM cells, including immune cells and tumor cells, especially how to mediate tumor invasion, we first designed a cellular hypoxia predicting framework, referred it as CHPF, to predict cellular hypoxia status and applied the CHPF to GBM single-cell RNA-seq data. Our work provided a landscape of cellular hypoxia and uncovered the critical regulators in hypoxic subpopulation with high invasive potential in GBM. The critical regulators could act as promising factors for the survival of patients and drug targets of hypoxia-targeted GBM treatments.

## Materials and Methods

### Single cell RNA-seq data acquisition and processing

We downloaded the single-cell RNA transcription data of GBM from the GSE84465 22, GSE117891 23, GSE125587 24, GSE131928 25 in the Gene Expression Omnibus (GEO) database, which included 3589 cells (four GBM patients form two separate locations, tumor core and surrounding peripheral tissues), 6148 cells (13 GBM patients collected from tumor core, tumor peripheral tissues and normal tissues), 21750 cells (ten GBM patients), and 16201 cells (nine GBM patients), respectively. The raw count expression profiles were normalized and scaled using the Seurat 26 R package and genes were removed if they expressed in < 0.1% cells.

### Identification of high-confidence cells

We used "hypoxia" and "hypoxic" as the searching terms in the Molecular Signature database (MSigDB) and screened out seven hypoxia-related gene sets by the following conditions: (1) obtained from human; (2) without knockout experiments or other chemical compounds; (3) up-regulated under hypoxia; (4) removed redundant gene sets. Single sample gene set enrichment analysis (ssGSEA) was used to calculate activity score of each cell in each hypoxia gene set 27. For one gene set, Gaussian mixture model (GMM) was used to assign cells into high- and low-score group based on cells' activity scores 28. Cells were considered as high-confidence hypoxic cells if they were all assigned to the high-score group in seven GMMs, and cells were considered as high-confidence normoxic cells if they were all assigned to the low-score group in seven GMMs.

### Cellular hypoxia predicting framework (CHPF)

We used high-confidence cells to predict the hypoxia status of other unclassified cells. Differential expression analysis between high-confidence hypoxic and normoxic cells was performed by wilcoxon rank sum test and the top 500 differential expressed genes (DEGs) were selected as input features. Five-fold cross validation was performed to assign the training set and the test set. In each training set, we obtained all hypoxic cells and the same size of normoxic cells by random sampling. Next, we used Light Gradient Boosting Machine (LightGBM) to build the Gradient boosting decision tree to classify cells into hypoxic or normoxic cells. This step will be performed 100 times and obtained 100 decision trees, 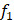, 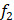,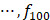. Given that the different accuracy of decision trees, the equal-weight voting ensemble classifiers cannot effectively reduce errors caused by inefficient sub-classifiers. Thus, for each decision tree, we used the test set to evaluate its effectiveness and assigned the recall rate as the weight of the decision tree. The voting rate formula was defined as following:



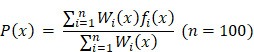



where 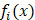 is the indicator function, representing the status of cell 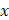 in the decision tree 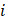. If the cell is predicted to be hypoxic, 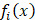 = 1, otherwise 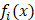 = 0. 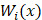 denotes the weight of each decision tree which is calculated by recall rate. The voting rate 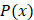 determines the final prediction result. If 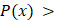
0.5, the cell will be assigned as a hypoxic cell, otherwise it is assigned as a normoxic cell.

Then, we selected genes that were used as features in more than 90% of decision trees, and performed differential expression analysis on them in each cell type to identify cell type-specific hypoxia-related gene signatures (Wilcoxon test, FDR < 0.05 and log2FC > 1.5).

### Cell-cell communication analysis

Cross-talks between tumor cells and immune cells in different hypoxia status were done using the CellChat package 29. The gene expression data of each cell was obtained from the Seurat normalized expression matrix. We identified overexpressed ligands or receptors among cell types, and used the “computeCommunProbPathway” function to infer the cell-cell communication at a signaling pathway level.

### Single cell RNA-seq clustering and marker genes identification

We applied the top 2000 high variable features of the dataset to perform principal component analysis for reducing the dimensionality of data, and the top 20 principal components were used for downstream analyses. The cell subpopulations were identified using the “FindClusters” function (resolution = 0.6) and visualized using UMAP 30. To identify the marker gene for each subpopulation, we used the “FindMarkers” function between each hypoxic (normoxic) subpopulation to all normoxic (hypoxic) tumor cells.

### Metabolic pathways and biological processes enrichment analysis

We obtained metabolic pathways and pathway-related genes from KEGG database. The activity scores of 80 metabolic pathways in each subpopulation (H1, H2, H3, N1, N2, N3) were calculated according to Xiao et al. 31. The input data was the normalized expression matrix.

The R package clusterProfiler was used to conduct GO biological process analysis 32. Go terms of adjusted p value < 0.05 were considered as significantly enrichment. Cytoscape plugins EnrichmentMap and AutoAnnotate were used to visualize similar pathways with stringent pathway similarity scores 33,34.

### Inference of copy number variation alterations

We used inferCNV to infer CNAs from single cell RNA sequencing data. To determine the distinct chromosomal copy number patterns of each subpopulation, we set oligodendrocyte progenitor cells as "reference" cells. The CNA score of each cell was calculated as a quadratic sum of CNVregion.

### Cell status-specific gene regulatory network analysis and critical genes identification

The cell status-specific gene regulatory networks were constructed for H2 subpopulation and N2 subpopulation by SCENIC, respectively 35. GENIE3 was used to construct a co-expression network and RcisTarget was used to identify regulons for each transcription factor 36. Then, we employed a centrality metrics method, as described in a previous study, to measure the importance of nodes 37. The method contained five centrality metrics, including degree, betweenness, eigenvalue, PageRank and closeness. Q statistic was used to integrate the five-centrality metrics of the nodes and the top 1% genes ranked by the integrated Q statistic in each network were considered as critical genes.

### Evaluation of associations between critical TFs and clinical outcomes

We downloaded two gene expression datasets from TCGA and GEO (GSE16011 38), which contained gene-expression data and clinical information. After filtering out patients without clinical information, 518 GBM patients from TCGA were treated as a training dataset, and 150 GBM patients from GSE16011 were used as an independent validation dataset. We performed the univariate Cox proportional hazards regression to investigate the association between the expression of critical TFs and the OS of GBM patients in the training set. Significant OS-related genes were selected (P < 0.05) to further perform variable selection using stepwise regression analysis. Ultimately, there were four TFs (*FOSL1*, *CEBPD*, *MXI1*, *YY1*) that were identified in our study to construct the final prognostic model based on the gene expression weighted by regression coefficients of univariable Cox regression analysis.



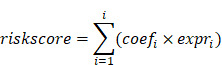



Patients were classified into the high-risk group and the low- risk group based on the median risk score. KM curves were used to compare the OS between two groups. Multivariate Cox regression analyses were performed with risk score, age, sex, Karnofsky score, Histological subtype, molecular subtype and IDH1 mutation as variables to assess whether risk score could be as an independent predictor.

### Sensitivity analysis of anticancer drugs and drug-perturbed analysis

We downloaded the drug sensitivity data and gene expression profiles of cancer cell lines from Genomics of Drug Sensitivity in Cancer (GDSC). First, we selected expression data of brain cancer cell lines and calculated Spearman's correlation between the expression of 14 critical TFs and the AUC of compounds (|cor| > 0.3 and FDR < 0.05). Then, we employed the recently updated LINCS (Expanded CMap LINCS Resource 2020), a data-driven, systematic approach for discovering correlations among genes, chemicals, and biological conditions, to search for candidate compounds that might target critical TFs we identified above. In this study, we focused on brain cancer cell lines and neuroblastoma cell lines, which included 6 cell lines and 906 compounds. With a strict requirement that the drug treatment concentration was 10 um, treatment time was 24 h, and the number of drugs in per cell line > 10, we finally obtained 1182 drug perturbation data. Then, based on the weighted Kolmogorov-Smirnov enrichment statistic (ES) we calculated the weighted connectivity score of each drug per cell line. WTCS ranges from -1 to 1. Greater than 0 means positive correlation, less than 0 means negative correlation, and near zero for signatures that are unrelated.

### Cell lines

All GBM cell lines (U87, LN229) were purchased from the Institute of Biochemistry and Cell Biology, Chinese Academy of Science. Patient-derived GBM cell line (HG9) was obtained from patients with primary GBM. U87, LN229, and HG9 cell lines were cultured in DMEM (Gibco) medium with 10% FBS (Gibco) and penicillin/streptomycin 25000U supplemented, and a humid atmosphere in 37℃ with 5% CO_2_. U87, LN229, and HG9 cells interfered with* CEBPD* and *FOSL1* siRNA and control vectors as indicated.

### 3D Sphere Invasion Assay

Our performance followed the manufacturer's instructions as indicated by 3D Spheroid BME Cell Invasion Assay Kit (Trevigen). Harvested 2000 cells for each well and washed them in PBS. Resuspended cells in spheroid formation ECM and added to 96-well spheroid formation plate. After incubating in a hypoxic incubator with 1% O_2_ and 5% CO_2_ for 2-3 days, added invasion matrix and culture medium with 20% FBS. Cells invaded the matrix and obtained images at different time points. All experiments were performed in triplicate.

### Transwell invasion assay

The transwell invasion assay was performed in 24-well plates with a 6.5 mm insert transwell chamber with 8 μm polycarbonate membrane (Corning) pre-coated Matrigel (Corning). The single cell suspension was added into upper chamber with 5×10^4^ cells in 200 μL culture medium with 2% FBS, and 500 μL culture medium with 20% FBS were added into the lower chamber. After incubating in a hypoxic incubator with 1% O_2_ and 5% CO_2_ for 48 to 72 h, discarded the solution in the upper chamber and wiped the upper layer of the membrane. Move the chamber into 4% PFA to fix for 5 min. Stain the membrane with crystal violet for 5 min. Finally, we obtained the photographs on the microscope. All experiments were performed in triplicate.

### Wound healing assay

Mark the back of the 6-well plate by marking a straight line, inoculate 5×10^5^ cells into the wells, and the next day, when the cell confluence reaches approximately 100%, score with a sterile tip perpendicular to the cell plane and the previously scored line. Then the cells were washed three times with sterile PBS buffer, replaced with medium containing 2% FBS, incubating in a hypoxic incubator with 1% O_2_ and 5% CO_2_ at 37℃, and then photographed and recorded and counted every 24 hours to measure the area of wound healing. All experiments were performed in triplicate.

### Mice Model

Briefly, 1×10^6^ U87 GBM cells were injected into 5-week-old female immunodeficient BALB/c Nude mice to establish a glioblastoma model in situ. Six mice were used in each group of experiments. Mice were grouped into 4 groups after tumor injection that were treated with DMSO, TMZ (60 mg/kg/day via intra-peritoneal injection in DMSO, purchased from Selleck) 39, Axitinib (30 mg/kg/day oral administration, purchased from Selleck) 40, and Entinostat (25 mg/kg/day oral administration, purchased from Selleck) for 7 days. Tumor volume was detected by luciferase via in vivo imaging using IVIS Spectrum CT (PerkinElmer).

## Results

### Characterization of the landscape of cell hypoxia in GBM

**Figure 1:**
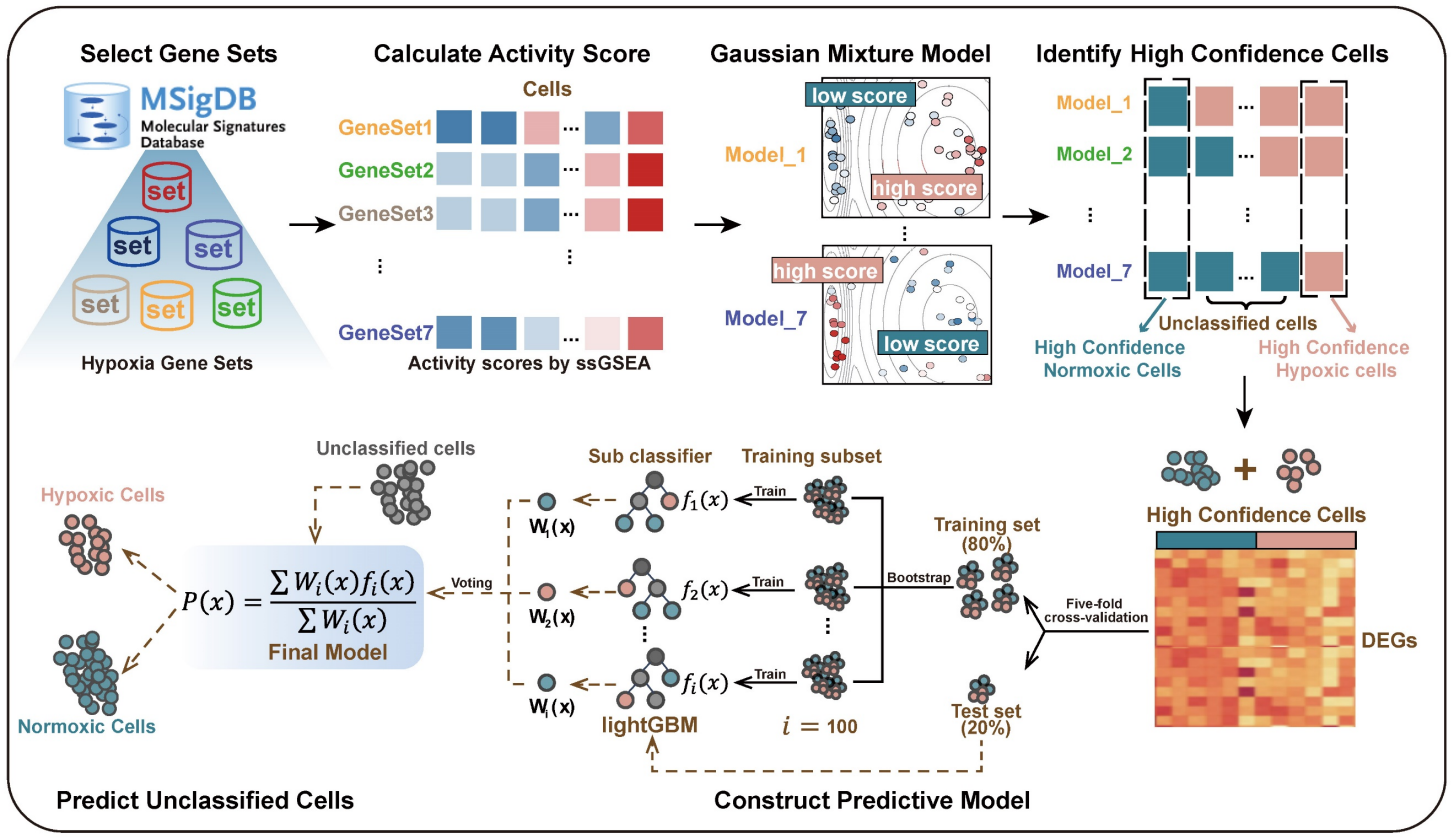
Overview of the cellular hypoxia predicting framework (CHPF).

GBM is characterized by extensive tissue hypoxia. To explore and understand the hypoxic landscape of GBM cells, we designed CHPF, a novel framework to classify cellular hypoxia status by integrating single cell transcriptome data and hypoxic gene signatures (Figure 1). Specifically, we screened out seven hypoxia gene signatures (Table S1), and calculated cells' activity scores in each gene signature. Then, in all seven GMMs, cells assigned to high (low) hypoxic score groups were considered as high-confidence hypoxic (normoxic) cells. These high-confidence cells were used to construct a cellular hypoxia status predictive model based on the LightGBM algorithm.

**Figure 2:**
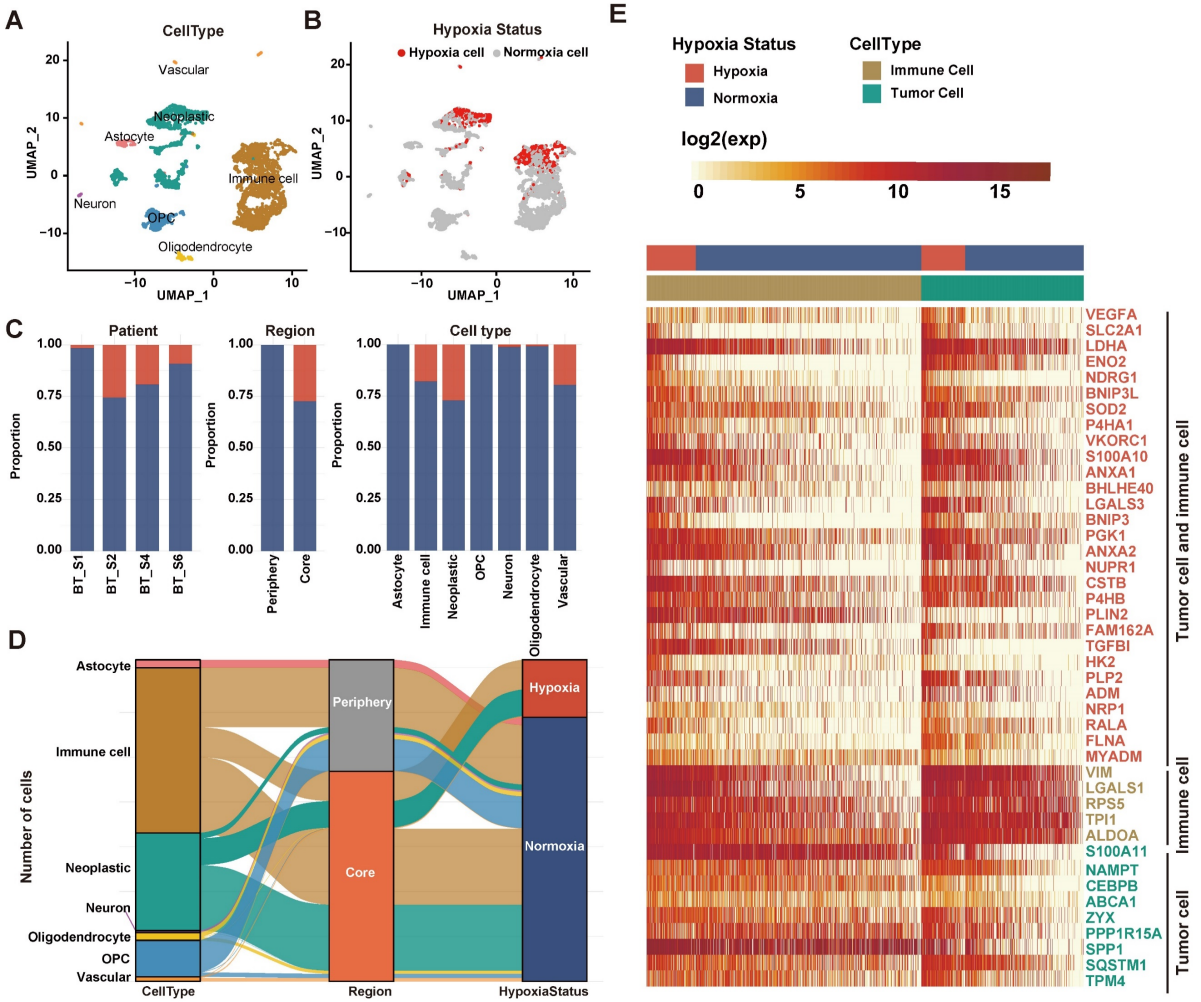
** Defining cellular hypoxia status in GBM.** (A) UMAP plot of all the 3,589 single cells in 4 primary GBMs. Cell types were differentiated by colors. (B) UMAP plot of all single cells colored by hypoxia status. (C) Distribution of hypoxic cells in different patients, regions and cell types. (D) Sankey diagram showing the connection of cell types, cell- originating regions and hypoxia status. (E) Expression profile of hypoxia-related genes. The 29 red-colored genes were identified as important hypoxia-related genes in both tumor cells and immune cells, the five brown-colored genes were only identified as important hypoxia-related genes in immune cells, and the nine green-colored genes were only identified as important hypoxia-related genes in tumor cells.

We applied the CHPF to a GBM dataset (GSE84465), which was collected from two separate locations, tumor core and surrounding peripheral tissues of four patients. The data comprised tumor cells and each of the major CNS cell types, such as vascular, immune, neuron and glial (Figure 2A). By employing the CHPF, we classified 642 cells as hypoxic cells and 2,947 cells as normoxic cells (Figure 2B). We observed hypoxic cells were mainly composed of neoplastic cells and immune cells, and the proportion of hypoxic cells varied among different patients (Figure 2C). As expected, hypoxic cells were all derived from the tumor core, which also demonstrated the reliability of our predictive framework (Figure 2D).

We screened important features in the framework that can distinguish hypoxia status (Table S2). Fifty important genes were identified, out of 43 were differentially expressed between hypoxia cells and normoxic cells in tumor cells and/or immune cells (Figure 2E). Among them, 29 genes as important hypoxia-related genes were in both tumor cells and immune cells, such as *VEGFA, SCL2A1, LDHA,* and* BHLHE40.* Five genes as important hypoxia-related genes were differentially expressed only in immune cells, including *VIM*, *LGALS1* and *RPS5*. Nine genes as important hypoxia-related genes were differentially expressed only in tumor cells, such as *SPP1*, *S100A11* etc. The majority of genes have been reported to be involved in various cancer-related processes induced by hypoxic microenvironment, including angiogenesis (*VEGFA*, *ADM*), glycolytic (*SLC2A1*, *LDHA*, *ENO2* and *HK2*), invasion (*S100A10*), chemoresistance (*NDRG1*) and autophagy (*BNIP3*).

Furthermore, using three additional large-scale GBM scRNA-seq datasets (GSE117891, GSE125587, GSE131928), we evaluated the performance of the CHPF. In each dataset, we first calculated the activity scores of cells in seven hypoxia gene signatures and identified high-confidence cells. The high-confidence cells were randomly assigned into the training set and testing set with a ratio of 2:1, and then we computed the difference between observed values and predicted values (Figure S1, Table S3). The result showed that our framework had high sensitivity and specificity in the testing set with an AUC of 0.9995, 0.969 and 0.992 for GSE117891, GSE125587 and GSE131928, respectively. And similarly, hypoxic cells were also all derived from the tumor core in GSE117891 (Figure S2).

### Exploring the cell-cell interactions between immune cells and tumor cells in hypoxic microenvironment

**Figure 3:**
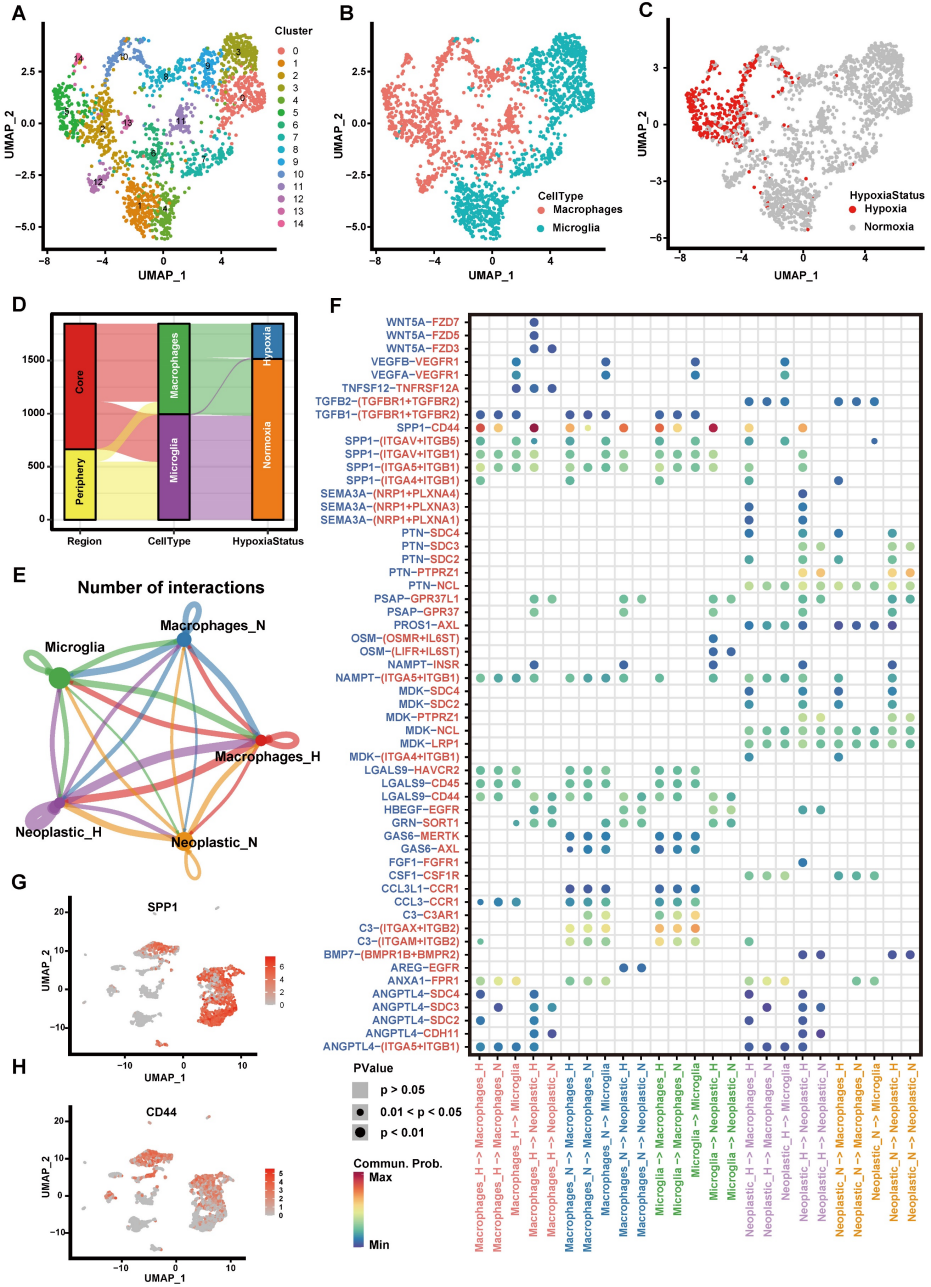
** Cell-cell communication between immune cells and tumor cells.** (A-C) The UMAP plot showing immune cells. (A) Different colors labeled for 15 clusters, respectively. (B) Different colors labeled for different cell types. (C) Different colors labeled for different hypoxia status. (D) Sankey diagram showing the connection of cell types, cell collected regions and hypoxia status in immune cells. (E) Cell-cell interaction network of hypoxic tumor cells, normoxic tumor cells, microglia, hypoxic macrophages, normoxic macrophages. The node size represents the number of interactions. The width of the edge represents the number of significant ligand-receptor interactions in two cell types. (F) Bubble heatmap showing cells interaction strength for different ligand-receptor pairs. Dot size indicates p-value generated by the permutation test and dot color represents communication probabilities. Empty space indicates that the communication probability is zero. (G-H) The UMAP plot showing the *SPP1* (G) and *CD44* (H) expression level in all cell types.

We first explored the hypoxia landscape on immune cells as it was one of the major cell types affected by hypoxia 41. We manually annotated immune cells using canonical markers and found that they were mainly composed of macrophages and microglia (>95%) (Figures 3A-B and Figure S3). Among them, hypoxic cells were mainly found in macrophages and rarely found in microglia, although both of them were partially derived from the tumor core (Figures 3C-D). It implied that macrophages were more susceptible to the changes of oxygen concentration in the microenvironment. It is consistent with the previous research that macrophages are the most abundant immune cells in the tumor microenvironment, which can accumulate in large numbers in hypoxic tumor areas and become a lethal combination with hypoxia 42, 43.

Next, we divided macrophages into hypoxic macrophages (Macrophages_H) and normoxic macrophages (Macrophages_N), and investigated the cell-cell communication between immune cells and tumor cells by modeling ligand-receptor interactions. Between various immune cell types and tumor cells, 325 pairs of interactions and 23 significant signaling pathways were identified (Figure 3E). Calculating the interaction strength for selected ligand-receptor pairs in distinct cell types, we inferred a cell state-specific ligand-receptor interaction network (Figure 3F). For example, tumor cells expressed relatively high levels of *SEMA3A*,* PTN* and *MDK* etc. Midkine (*MDK*), a heparin-binding growth factor that can promote tumor cell proliferation and EMT 44, while the corresponding receptors (*SDC4*, *SDC2*) were widely expressed in hypoxic cells (Macrophages_H, Neoplastic_H). It suggested that these ligands played significant roles in influencing immune cell infiltration in hypoxic microenvironment.

Notably, interaction of *SPP1-CD44* has high interaction scores across multiple cell types (Figure 3F). By computing multiple network-centric measurements per cell type, it showed that* SPP1* signal was mainly sent by Macrophages_H and received by Neoplastic_H (Figure S4). We also observed that *SPP1* gene mainly expressed in immune cells (Figure 3G), whereas the *CD44* mainly expressed in tumor cells (Figure 3H). Most previous studies have shown that the increased *SPP1* expression induces GBM-associated macrophage infiltration and is associated with poor prognosis in GBM patients 45-47. Meanwhile, hypoxia not only directly affects macrophage polarization, but can have indirect effects by altering the communication between tumor cells and macrophages 42, 48, 49. Our findings suggested that *SPP1-CD44* interaction in hypoxic microenvironment may play a critical role in activating tumor cells to produce a more malignant phenotype.

### Deciphering the heterogeneity of hypoxia status and functions in GBM tumor cells

**Figure 4:**
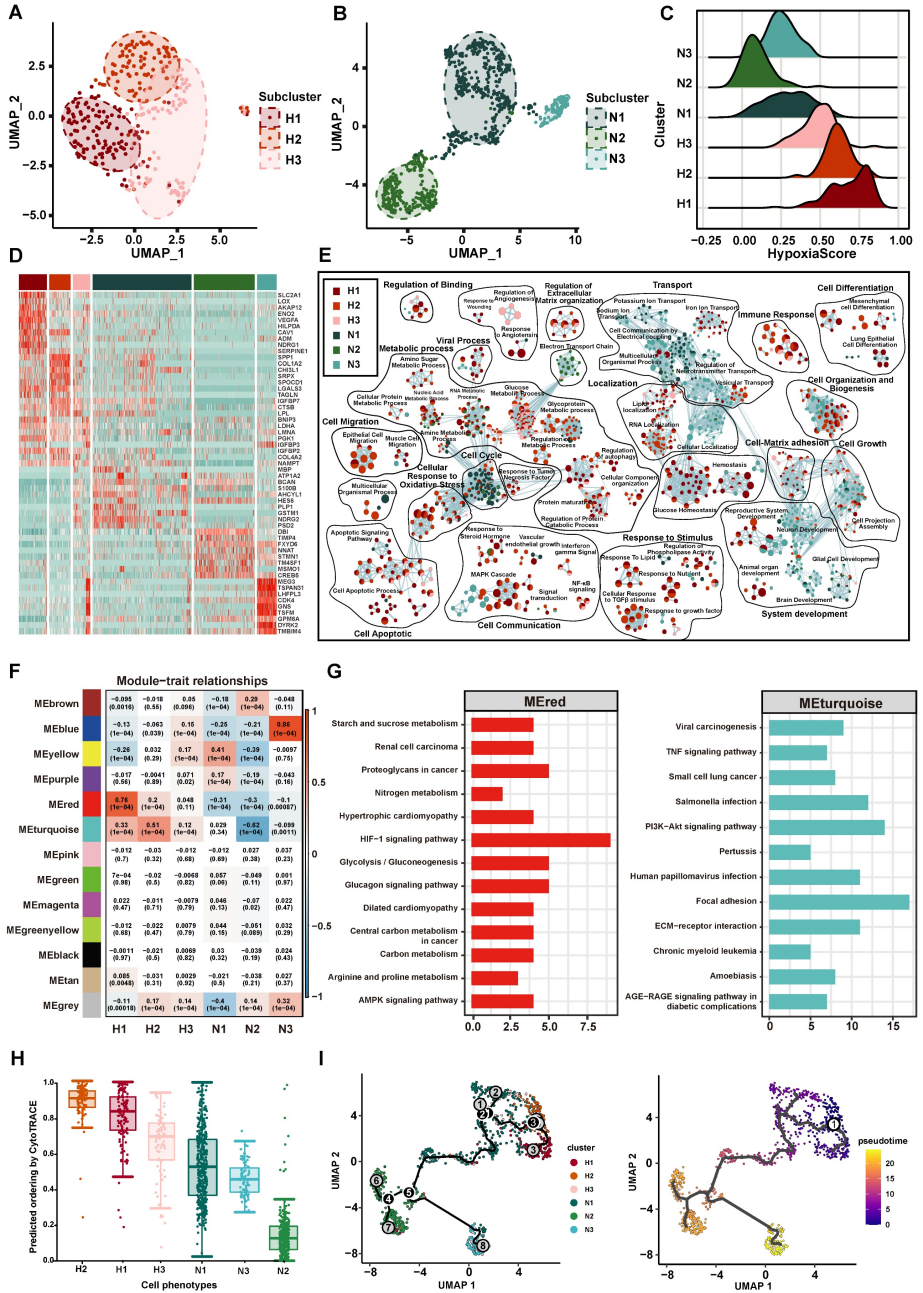
** The Characteristics of tumor cell subpopulation.** (A-B) The UMAP plot showing hypoxic tumor cell subpopulations (A) and normoxic tumor cell subpopulations (B). Cell subpopulations are differentiated by colors. (C) Density distribution of hypoxia scores in six tumor cell subpopulations. (D) The heatmap plot of the top 10 differentially expressed genes in each subpopulation. (E) Enrichment map of biological pathways by marker genes in each subpopulation. Nodes in the network represent pathways and are colored by associated subpopulations. (F) The relationship between gene modules and subpopulations by WGCNA. (G) KEGG analysis of the MEred module (left) and MEturquoise module (right). (H) CytoTRACE score of each subpopulation. (I) Trajectory analysis of tumor cells, colored by each subpopulation (left) and pseudotime (right).

With the goal of characterizing the heterogeneity of hypoxia status of tumor cells in GBM, we first classified 1091 tumor cells into 296 (27.13%) hypoxic cells and 795 (72.87%) normoxic cells by CHPF. Compared to normoxic tumor cells, a higher correlation was shown among hypoxic tumor cells (Figure S5A). We performed clustering analysis on hypoxic and normoxic tumor cells, respectively and identified three hypoxic subpopulations (named H1, H2, H3; Figure 4A) and three normoxic subpopulations (named N1, N2, N3; Figure 4B). A “hypoxia score” was calculated for each cell on the basis of the expression of 38 important hypoxic genes in response to tumor cells identified above (Figure 4C). H1 subpopulation had the highest hypoxia score, revealing cells of H1 subpopulation had the lowest oxygen level (Figures S5B-D).

Then, we identified the unique gene expression patterns of these six subpopulations (Figure 4D, Table S4). Interestingly, the marker genes in the hypoxic subpopulations were previously shown to be associated with tumor progression, such as *SLC2A1* and *VEGFA* in H1 subpopulation. *SLC2A1* gene encodes the glucose transporter 1 protein, this protein is a membrane channel protein that is expressed on the cell membrane and transports glucose from the bloodstream into the brain and other tissues 50. The protein encoded by *VEGFA* is a cytokine that induces angiogenesis and increases vascular permeability, playing a critical role in the proliferation and survival of endothelial cells and other cell types 51. In addition, we found *SPP1* and *COL1A2* had high expression in H2 subpopulation, which participated in maintenance of the extracellular matrix. The GO function enrichment analysis revealed the marker genes of hypoxic subpopulations involved in cellular response to oxidative stress, cell apoptotic, cell migration, cell-matrix adhesion and cell differentiation (Figure 4E). Of note, markers of H2 subpopulation were enriched in tumor invasion-related pathways, such as cellular response to TGFβ stimulus, epithelial cell migration and RNA localization. Normoxic subpopulations were characterized by an enrichment of neuron-related biological pathways, such as neuron development, cellular localization and ion transport.

Besides, we sought to identify key gene modules by WGCNA for each subpopulation (Figure 4F, Table S5). H1 subpopulation was highly associated with the MEred module (cor = 0.76, p < 1.0×10^-4^), and H2 subpopulation was highly associated with the MEturquoise module (cor = 0.51, p < 1.0×10^-4^). The key genes of MEred modules (related to H1 subpopulation) were enriched in response to hypoxia, HIF-1 signaling pathway and glycolysis/gluconeogenesis, and the key genes of MEturquoise module (related to H2 subpopulation) were enriched in cell adhesion, PI3K-Akt signaling pathway and TNF signaling pathway (Figure 4G). Through metabolic pathway activity analysis 31, we also found that H1 had a high metabolic activity score in Glycolysis/Gluconeogenesis pathway and H2 enriched in Steroid hormone biosynthesis (Figure S6). Taken together, our results provided an overview of the functional heterogeneity of tumor cells with different hypoxia status in GBM.

In addition, we explored the correlation between the hypoxia status and the cell differentiation. Using CytoTRACE to calculate the differentiation status of each tumor subpopulation, we found that the H2 subpopulation had the highest differentiation score (Figure 4H). Subsequent trajectory analysis revealed that tumor cells with high hypoxia scores were positioned at the beginning of the pseudotime trajectory (Figure 4I).

### Construction of cell status-specific gene regulatory networks and identification of critical regulators in hypoxic-induced tumor invasion

**Figure 5:**
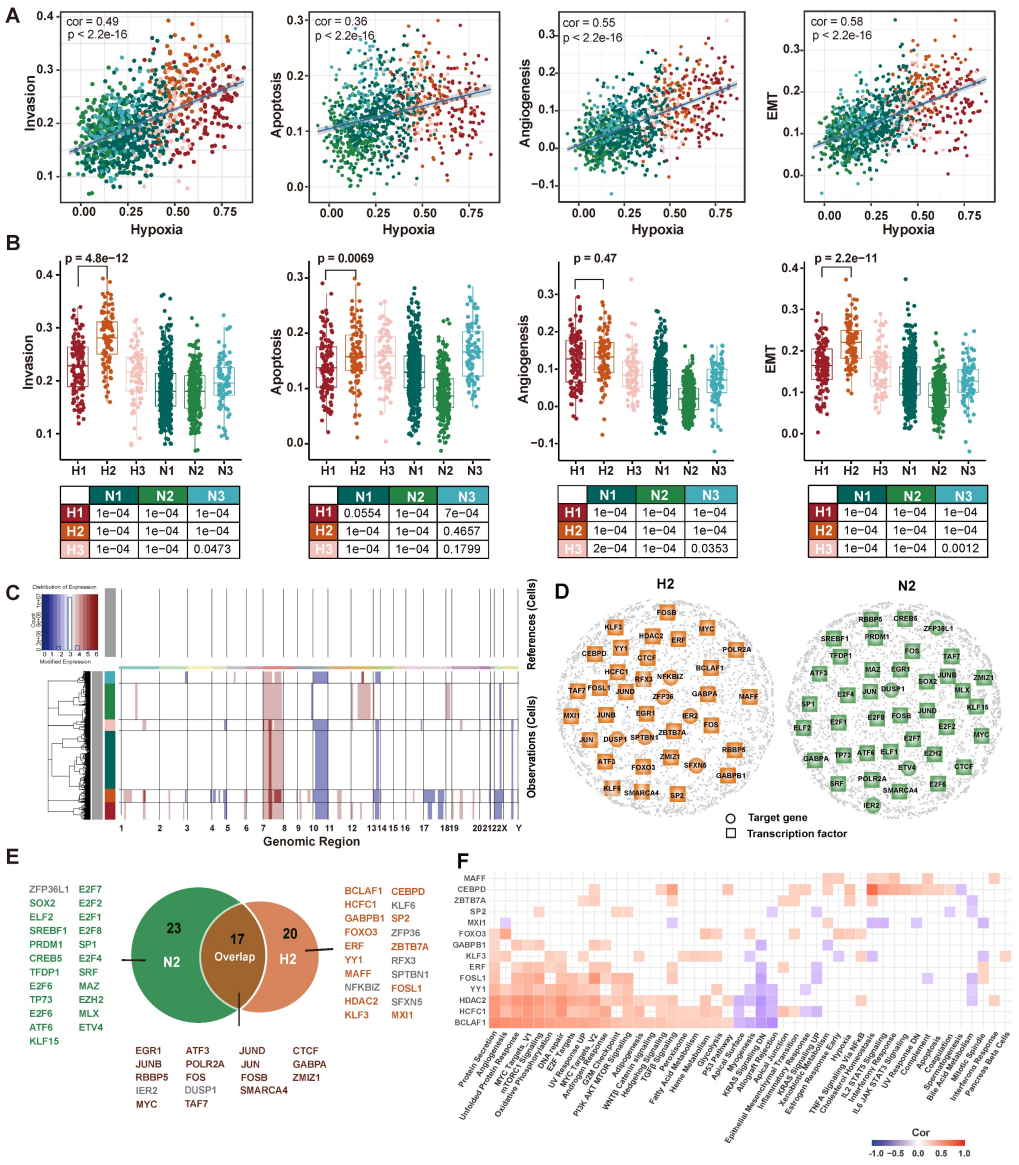
** Transcriptomic and genomic analysis of hypoxia and invasion.** (A) Correlation analysis of hypoxia with invasion, apoptosis, angiogenesis, and EMT. (B) Boxplot showing the invasion, apoptotic, angiogenesis, and EMT enrichment scores for each subpopulation. (C) Heatmap of the inferred CNAs across six tumor subpopulation cells, in which genes are sorted by genomic location. (D) GRNs of H2 subpopulation (left) and N2 subpopulation (right). Colored nodes imply the critical target genes (circle) or TFs (square). (E) The critical TFs and critical target genes identified from two GRNs. Green represents TFs recognized only in the N2 GRN, orange represents TFs recognized only in the H2 GRN, and brown represents TFs recognized in both GRNs. Gray represents target genes recognized in GRNs. (F) Correlation analysis between hallmark pathway enrichment scores and regulatory module enrichment scores. The enrichment scores were calculated by GSVA. Empty space indicates that the |cor| < 0.3 or p >0.05.

The cells have been found to evolve toward an aggressive tumor phenotype under hypoxic conditions in functional analysis. Therefore, we further explored the association between hypoxia and tumor invasion in GBM. We obtained signature genes associated with hypoxia, invasion, apoptosis, angiogenesis and EMT from CancerSEA database 52 (Table S6), and then calculated the activity score of each cell subpopulation for each cell by GSVA 53. The results showed that tumor invasion was significantly positively correlated with hypoxia (Figure 5A). Interestingly, H2 subpopulation had a higher invasive potential than H1 subpopulation (p = 4.8×10^-12^, Figure 5B). Furthermore, given that copy number alterations (CNAs) were hallmarks of malignant cells, we inferred the CNAs of tumor cells in the six subpopulations, respectively (Figure 5C). The loss of 10 and 17 chromosomes and the gain of 1 and 7 chromosomes were observed in H2 subpopulation, and it had significantly higher CNAs scores than other subpopulations (Figure S7). Collectively, our results highlighted that H2 subpopulation cells represented a hypoxic phenotype with highly invasive potential by transcriptome and genomic analysis.

To further elucidate the regulatory mechanisms of hypoxia-induced tumor cells to be more aggressive, cell status-specific GRNs were constructed by SCENIC in H2 subpopulation and N2 subpopulation (as reference), respectively. We then identified the critical genes in each GRN using centrality metrics of the network, including degree, PageRank, betweenness, eigenvalue, and closeness 37. The Q statistic was used to integrate the five-centrality metrics and top 1% genes ranked by the Q statistic were considered as critical genes 54 (Figure 5D). A total of 37 and 40 critical genes were identified in H2 and N2 subpopulation, respectively. Among 37 critical genes in the H2 subpopulation, 14 transcriptional factors were specific to H2 subpopulation (Figure 5E). They were considered to play an important regulatory role in hypoxic-induced tumor invasion in GBM.

Next, we employed MSigDB hallmark gene sets to assess the cancer-related pathways involved in 14 TFs and their target genes (regulons). Most of them were positively correlated with hallmarks, such as *BCLAF1*, *HCFC1*, and *HDAC2* regulons (Figure 5F). *BCLAF1*, which has been proved to be a direct target of *HIF-1* and plays a crucial role in the regulation of *HIF-1α* stability under hypoxia 55. Knockdown of *BCLAF1* can significantly reduce hypoxia-induced *HIF-1α* expression 56. Meanwhile, we investigated that about half of the regulons were correlated with *MYC* targets. *MYC* as a potent oncogene contributes to malignancy by various mechanisms, which is a compelling therapeutic target in glioblastoma 57. Several studies have reported *MYC* is associated with tumor hypoxia, aggressiveness and metastasis 58, 59.

### Critical TFs of hypoxic-induced tumor invasion can predict clinical outcomes

To determine whether 14 critical TFs can contribute to clinical therapy and guide GBM prognosis, we performed survival analysis using a TCGA GBM cohort containing 518 patients as the training set. We focused on six TFs that were associated with OS by univariate Cox proportional hazards regression (log-rank test, p < 0.05, Table S7). Then, we further selected genes associated with OS by stepwise regression analysis and four TFs (*FOSL1*, *CEBPD*, *MXI1*, *YY1*) were identified to construct the final prognostic model. We calculated each patient's risk score based on the four TFs' expression levels weighted by their regression coefficients in the univariate Cox proportional hazards regression analysis.

**Figure 6:**
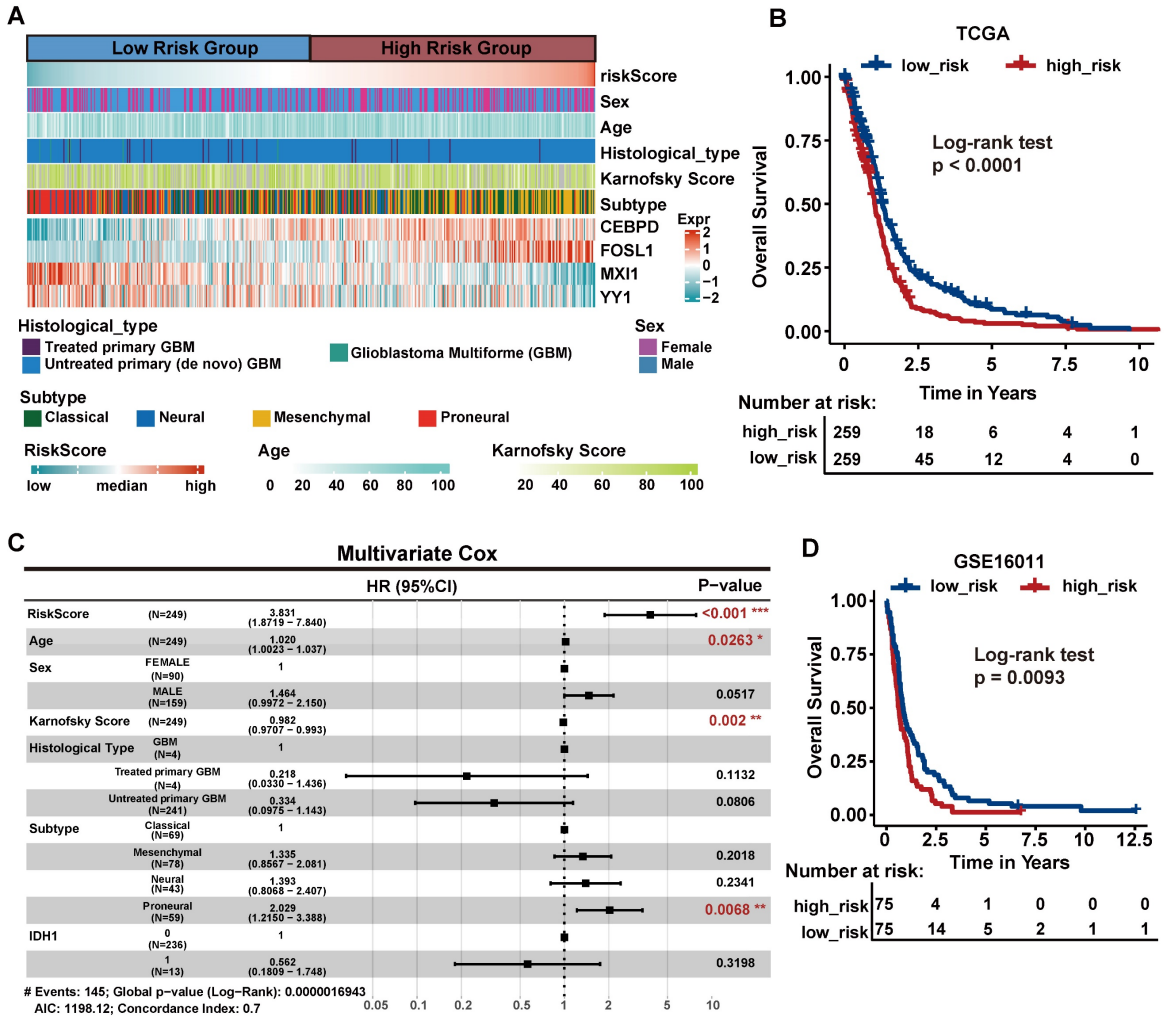
** The survival analysis of critical TFs.** (A) Heatmap of the expression of the four critical TFs in 518 GBM patients from TCGA cohort with clinical and histopathological characteristics. Patients were sorted by risk score. (B) Survival analysis based on the prognostic model in the TCGA cohort. Patients in the high-risk group had poor survival. Log-rank p < 0.0001. (C) Multivariate Cox regression analysis validated risk score as an independent prognostic factor in TCGA. (D) Survival analysis in an external validation set GSE16011.

We divided patients into the high-risk group (n = 259) and the low-risk group (n=259) according to the median risk score. We observed that the Proneural (PN) subtype patients were prone to have lower risk, whereas the Mesenchymal (MES) subtype patients had higher risk (Figure 6A, chi-square test p = 1.6
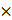
10^-17^). This finding is consistent with the previous study that MES subtype is more aggressive and strongly associated with a poor prognosis compared to PN subtype 60. The KM curves demonstrated that the high-risk group had significantly shorter OS than the low-risk group (log-rank test p < 0.001; Figure 6B). Next, multivariate Cox regression analysis was performed to assess whether the 4-TF signature was an independent prognostic factor (Figure 6C). The covariables included age, sex, Karnofsky score, histological type, subtype and IDH1 status. The result revealed that our 4-TF signature can independently predict a worse OS for GBM patients (HR = 3.528, 95%CI [2.2396-5.559], p < 0.0001). Finally, we validated the prognostic value of the 4-TF signature in an external validation dataset (GSE16011). The KM curves of the two groups were significantly different (p = 0.0093, Figure 6D).

### Potential drugs for hypoxia-targeted therapy in GBM

**Figure 7:**
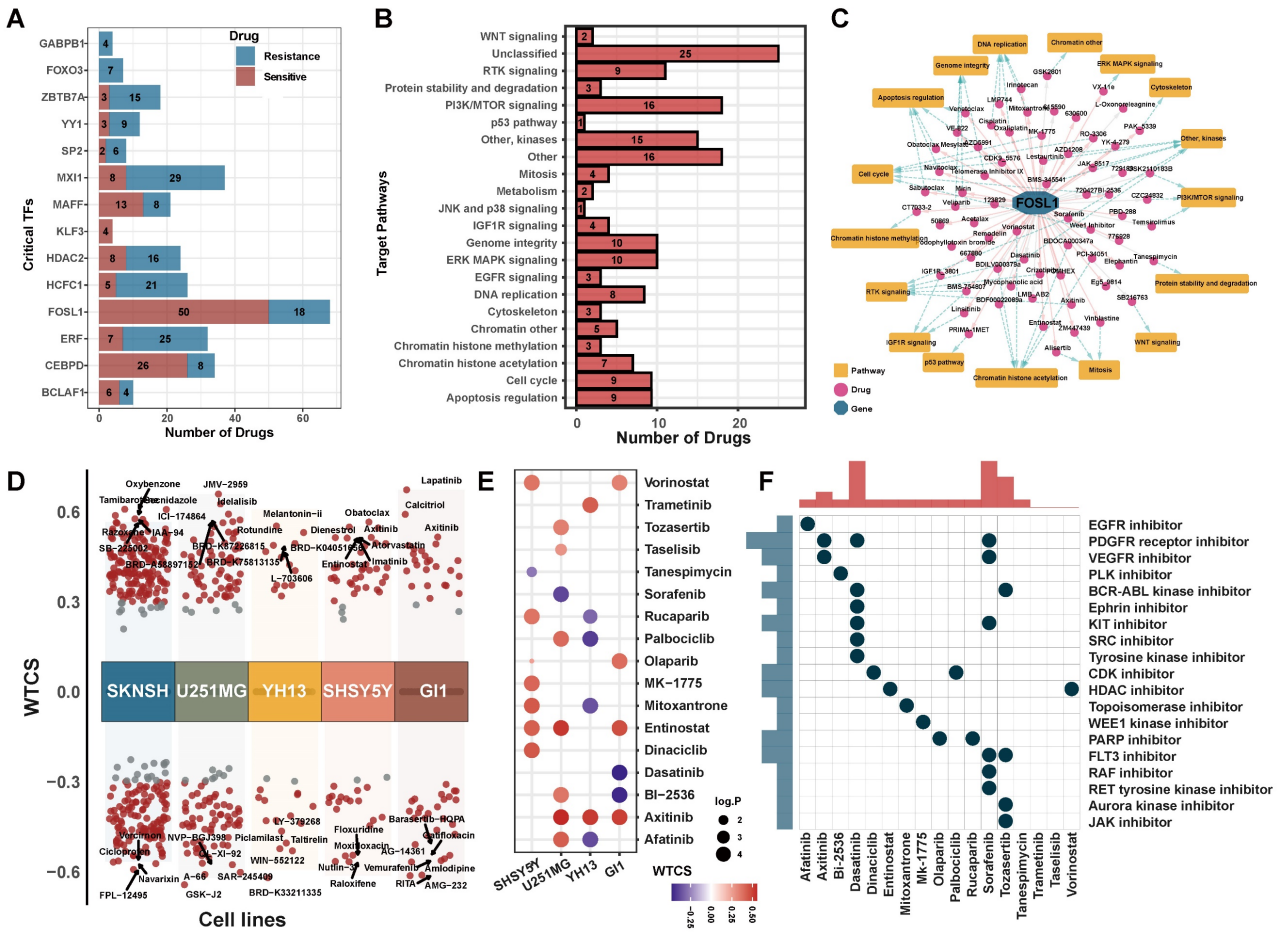
** Potential drugs targeting critical regulators.** (A) The number of potential drugs targeting each critical TF. (B) Signaling pathways targeted by predicted potential drugs. (C) The network of *FOSL1*-related drugs and signaling pathways. The pink line indicated a positive correlation and the gray line indicated a negative correlation. Blue dotted line indicated the relationship between drug and regulated signaling pathways. (D) Candidate drugs perturbed by DEGs of critical TFs targeted in five brain cancer cell lines. WTCS > 0 represented that the drug was positive to the gene signature, WTCS < 0 represented that the drug was negative to the gene signature, and near zero represented unrelated. (E) WTCS of 17 potential drugs identified in both two strategies. The dot color indicated the value of WTCS and the dot size indicated the significance level (-log10 P-value). (F) CMap mode of action (MoA) analysis of the 17 compounds.

To characterize the clinically applicable therapeutic implications of 14 critical TFs, we calculated Spearman's correlation coefficients between the expression of critical TFs and drug sensitive AUC of 286 anti-cancer compounds across 73 brain cancer cell lines from the GDSC (Table S8). We observed that each critical TF was significantly associated with at least four compounds (|cor| > 0.3, p < 0.05, Figure 7A, Table S9). These compounds were involved in multiple biological processes, including WNT signaling, PI3K/MTOR signaling and apoptosis signaling pathways (Figure 7B, Figure S8A). For example, the expression of* FOSL1* was linked to drug sensitivity of 50 compounds and drug resistance of 18 compounds (Figure 7C), including Wee1 Inhibitor, Entinostat and Axitinib.

Meanwhile, we used an alternative strategy to predict potential drugs aiding hypoxia-targeted therapy. By applying differential expression analysis to critical TFs and their target genes between H2 subpopulation and N2 subpopulation (Figure S8B), we identified 36 up-regulated genes and 4 down-regulated genes. These differentially expressed genes were considered as a gene regulatory signature and queried them against the LINCS database. We calculated the connectivity scores between compounds and the gene signature in five brain cancer cell lines. On average, more than 100 potential compounds were identified in each cell line (Figure 7D, Figure S8C).

Finally, a total of 17 compounds were determined in both two strategies (Figures 7E-F). Such as, Axitinib, an oral VEGFR and kinase inhibitor, showed a significant positively correlated with the signature in brain cancer cell lines. Benzamide histone deacetylase inhibitors (HDAC inhibitors), Entinostat and Vorinostat, also exhibited a positive correlation in cell line GI1, U251MG, and SHSY5Y (FDR < 0.01, WTCS > 0.5). It suggested that these compounds could be favorable candidates for future hypoxia-targeted therapy.

### Validation of the effects of regulators and potential drugs

**Figure 8:**
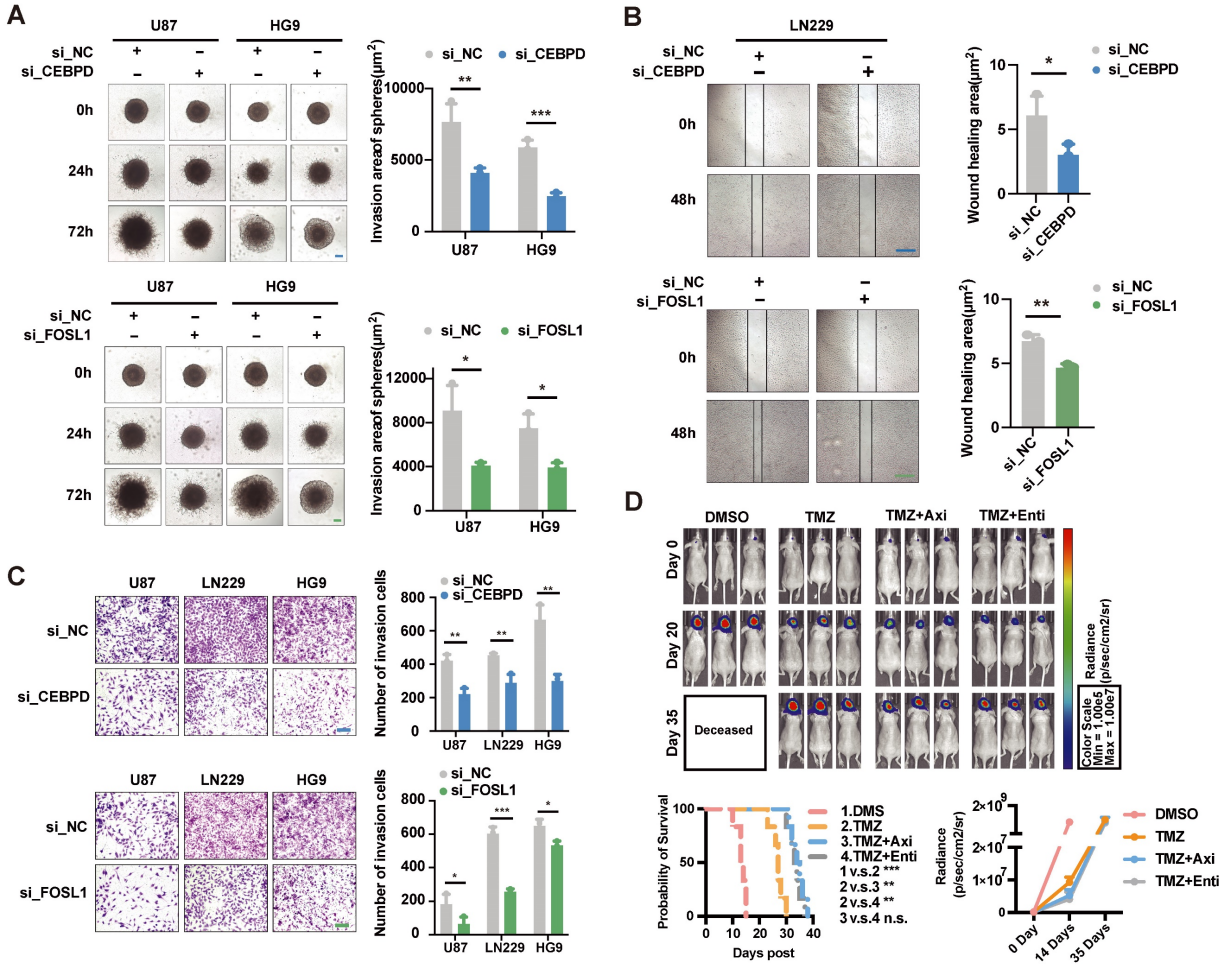
** Experimental validation of the effect of critical TFs and potential drugs in the hypoxia microenvironment.** (A) Effects on tumor cell invasion ability after interfered *CEBPD* and *FOSL1* with siRNA were examined by 3D sphere invasion assay in U87 and HG9 GBM cells under hypoxic conditions. Scale bar is 20 μm. Student's t-test, * *P* < 0.05, ** *P* < 0.01, *** *P* < 0.001. (B) The effect on tumor cell migration ability after interfered the *CEBPD* and *FOSL1* with siRNA was examined by wound healing assay in LN229 GBM cells under hypoxic conditions. Scale bar is 50 μm. Student's t-test, * *P* < 0.05, ** *P* < 0.01, *** *P* < 0.001. (C) Effects on tumor cell invasion ability by Transwell invasion assay in U87, LN229 and HG9 GBM cells after interference of *CEBPD* and *FOSL1* with siRNA under hypoxic conditions. Scale bars are 200 μm. Student's t-test, * *P* < 0.05, ** *P* < 0.01, *** *P* < 0.001. (D) Representative pictures and statistical plots of intracranial tumor size in mice after group treatment were shown by luciferase live imaging in an *in situ* tumorigenic mouse model constructed by U87 GBM cells, as well as statistics and analysis of survival in mice. Student's t-test and Log-rank (Mantel-Cox) test, * *P* < 0.05, ** *P* < 0.01, *** *P* < 0.001.

Based on the above analysis, *CEBPD* and *FOSL1* were identified as critical TFs involved in hypoxia-induced invasion, and both of them had high expression in the high-risk group patients. Therefore, we cultured three cell lines (U87, HG9, LN229) and designed the knockdown experiments to validate the effect of critical TFs in *in vitro* assay. The results showed that knocking down the expression of *CEBPD* and *FOSL1* significantly reduced cells' invasive ability under hypoxic conditions (Figures 8A-C), implying an important role of *CEBPD* and *FOSL1* in hypoxia-induced tumor invasion.

Meanwhile, we screened two small molecule inhibitors, Axitinib and Entinostat, targeting both *CEBPD* and *FOSL1*, and investigated their effects in mice models. Intracranial tumorigenic mice model *in situ* was constructed using the U87 GBM cell line, with the treatment of control (DMSO), TMZ, and combination of Axitinib or Entinostat with TMZ. The combination treatment of TMZ with Axitinib or Entinostat significantly improved the survival of mice and delayed tumor growth to some extent (Figure 8D). These results suggested that we might use Axitinib or Entinostat for adjuvant therapy to inhibit tumor growth and improve the survival of GBM patients, which had potential clinical translational prospects.

## Discussion

Hypoxia, a critical driver for cancer malignancy, is associated with poor prognosis. In this study, we designed a novel framework (CHPF) to define cellular hypoxia status and generated a hypoxia gene signature as a robust benchmark. Cell-cell communication analysis revealed that hypoxia influenced the crosstalk between macrophages and tumor cells via a variety of ligands and receptors. The heterogeneity of hypoxia status was observed and it associated with phenotype heterogeneity, including EMT, invasion, angiogenesis and apoptosis. One notable finding of this study was that we deciphered a hypoxic subpopulation with highly invasive potential (H2 subpopulation) and identified 14 TFs involved in tumor cell invasion induced by hypoxia at the single-cell resolution. The drugs predicted by us could act as a potential therapeutic approach to improve hypoxia-induced tumor invasion.

Many previous studies evaluated tumor hypoxia through calculating the activity scores of tumors in a specific hypoxia gene signature. However, hypoxia gene signatures are mostly obtained from different experimental conditions, which leads to a certain bias in defining cellular hypoxia status. Therefore, in CHPF, we first screened out hypoxia gene signatures with good performance and then identified high-confidence hypoxic and normoxic cells based on the consistency of their classification of hypoxia status. The differential expressed genes between two classes of high-confidence cells were used as features to build a classifier model to predict hypoxia status of the remaining unclassified cells. In addition, we used the undersampling method to balance the uneven high-confidence cell data. By validating from a 5-fold cross and external datasets, it was shown that the CHPF had a high sensitivity and specificity.

Several previous studies indicate that hypoxia stimulates complex signaling networks in tumor cells, including the HIF, PI3K, MAPK, and NFκB pathways, which are involved in cell apoptosis, migration and invasion 6, 13, 14. As expected, our results also demonstrated that hypoxia was significantly positively associated with invasion, apoptosis, EMT and angiogenesis. However, the activity scores of EMT and invasion of H2 subpopulation were higher than H1 subpopulation, although H1 had higher hypoxia score. These results suggested that hypoxia and invasion-related characteristics were not completely linear. We speculated that the cells of H1 subpopulation might be located in the most hypoxic regions. Rather than gaining the ability to invade other areas, the problem that these cells urgently need to solve is survival by inducing cells to undergo anaerobic glycolysis.

We identified 14 critical TFs specific to H2 subpopulation that may play an important regulatory role in hypoxia-induced tumor cell invasion. Especially, 4-TF signature that can predict the survival of GBM patients. In vitro experiments, we confirmed that knockdown of *CEBPD* and *FOSL1* affected cells' invasive ability. *YY1*, an important negative regulator of the tumor suppressor factor p53, researchers discovered that inhibition of *YY1* reduced the accumulation of HIF-1α and its activity under hypoxic conditions 61. Additionally, it has been reported that the protein encoded by *MXI1* is a transcriptional repressor thought to negatively regulate MYC function 62, and is therefore a potential tumor suppressor. Interestingly, in the hallmark analysis, we also found that *MXI1* was negatively correlated with angiogenesis, TGFβ signaling, and TNFA signaling via NFκB.

Our framework allows the prediction of cellular hypoxia status in large-scale scRNA-seq data, helping to dissect how the hypoxic microenvironment affects tumor cell invasion in glioblastoma. However, our study had several limitations. The landscape of hypoxia status in GBM was still preliminary, more cells from different tumor regions, or spatial transcriptomic data will help validate our framework and findings. As invasion is a very complex process, the regulatory mechanisms of the critical transcription factors we identified need further experimental confirmation.

In conclusion, we developed the CHPF to define cellular hypoxia status and elucidated the intratumor heterogeneity of hypoxia status in GBM. Of note, we dissected a distinct hypoxic subpopulation with high invasion potential, which had different transcriptome and genomic alterations. Our results suggested that *FOSL1*, *CEBPD*, *MXI1* and *YY1* were critical TFs of tumor invasion induced by hypoxia, and they can be used as an independent prognostic signature to predict GBM survival. The invasive ability of *CEBPD* and *FOSL1* was confirmed in vitro experiments. Mice tumor models revealed the effectiveness of Axitinib and Entinostat, two small molecule inhibitors targeting *CEBPD* and *FOSL1* against tumors. In total, our findings provided an advanced understanding of molecular characteristics and mechanisms of hypoxia-associated GBM invasion.

## Supplementary Material

Supplementary figures and tables.
