# Supplementary material for: Single-cell RNA sequencing identifies critical transcription factors of tumor cell invasion induced by hypoxia microenvironment in glioblastoma
Source: Theranostics. 2023 Jun 26;13(11):3744–60. doi: 10.7150/thno.81407 (PMC10334835; doi:10.7150/thno.81407)
Supplement: Supplementary file 1 — Supplementary figures and tables. [file thnov13p3744s1.zip › thno_81407r2_2.docx]

**Single-cell RNA sequencing identifies critical transcription factors of tumor cell invasion induced by hypoxia microenvironment in glioblastoma**

Yanru Zhang^1,#^, Bo Zhang^2,#^, Chengqian Lv^3,#^, Nan Zhang^4,#^, Kaiyuan Xing^1^, Zixuan Wang^1^, Rongkai Lv^1^, Mingchen Yu^5,*^, Chaohan Xu^1,*^, Yihan Wang^1,*^

^1^ College of Bioinformatics Science and Technology, Harbin Medical University,

Harbin, 150081, China.

^2^ Department of Pharmacology, State-Province Key Laboratories of Biomedicine-Pharmaceutics of China, Key Laboratory of Cardiovascular Medicine Research, Ministry of Education, College of Pharmacy, Harbin Medical University, Harbin, 150081, China.

^3^ Department of Gastroenterology and Hepatology, The Second Affiliated Hospital of Harbin Medical University, Harbin, 150086, China

^4^ College of Life Science and Technology, Huazhong University of Science and Technology, China.

^5^ Beijing Neurosurgical Institute, Capital Medical University. Beijing 100069, China.

^#^ These authors contributed equally.

^*^ Corresponding authors:

Yihan Wang, College of Bioinformatics Science and Technology, Harbin Medical University, Email: wangyihan@hrbmu.edu.cn;

Chaohan Xu, College of Bioinformatics Science and Technology, Harbin Medical University, Email: chaohanxu@hrbmu.edu.cn;

Mingchen Yu, Beijing Neurosurgical Institute, Capital Medical University. Email: yumc@mail.ccmu.edu.cn


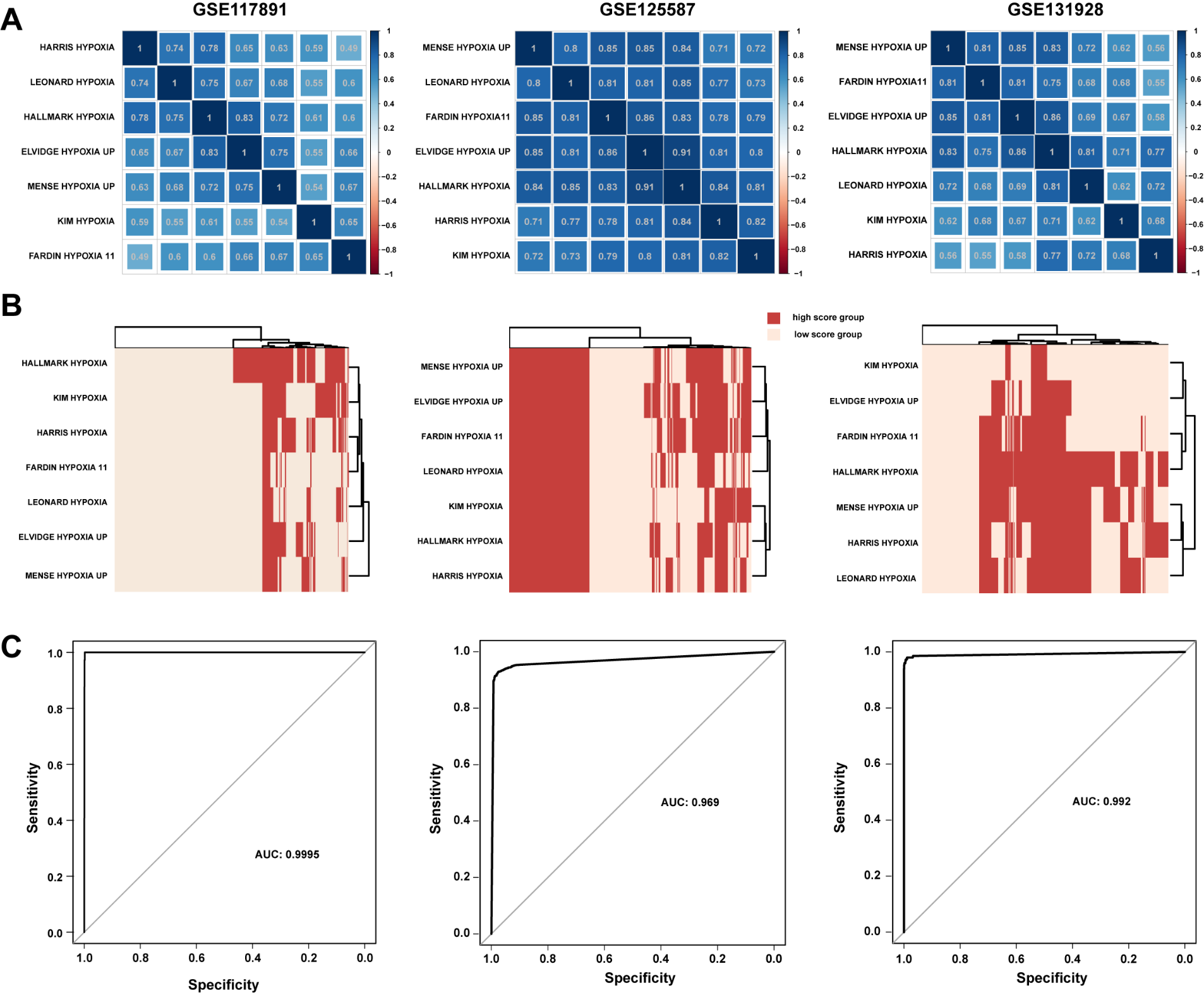


**Supplementary Figure S1 Evaluation of CHPF performance.** (A) Correlations between hypoxia scores for seven gene sets in GSE117891 (left), GSE125587 (median), and GSE131928 (right). (B) Cells of high (low) hypoxic score group in different dataset of GSE117891 (left), GSE125587 (median), and GSE131928 (right). (C) Performance evaluation of the CHPF in different datasets about GSE117891 (left), GSE125587 (median), and GSE131928 (right).


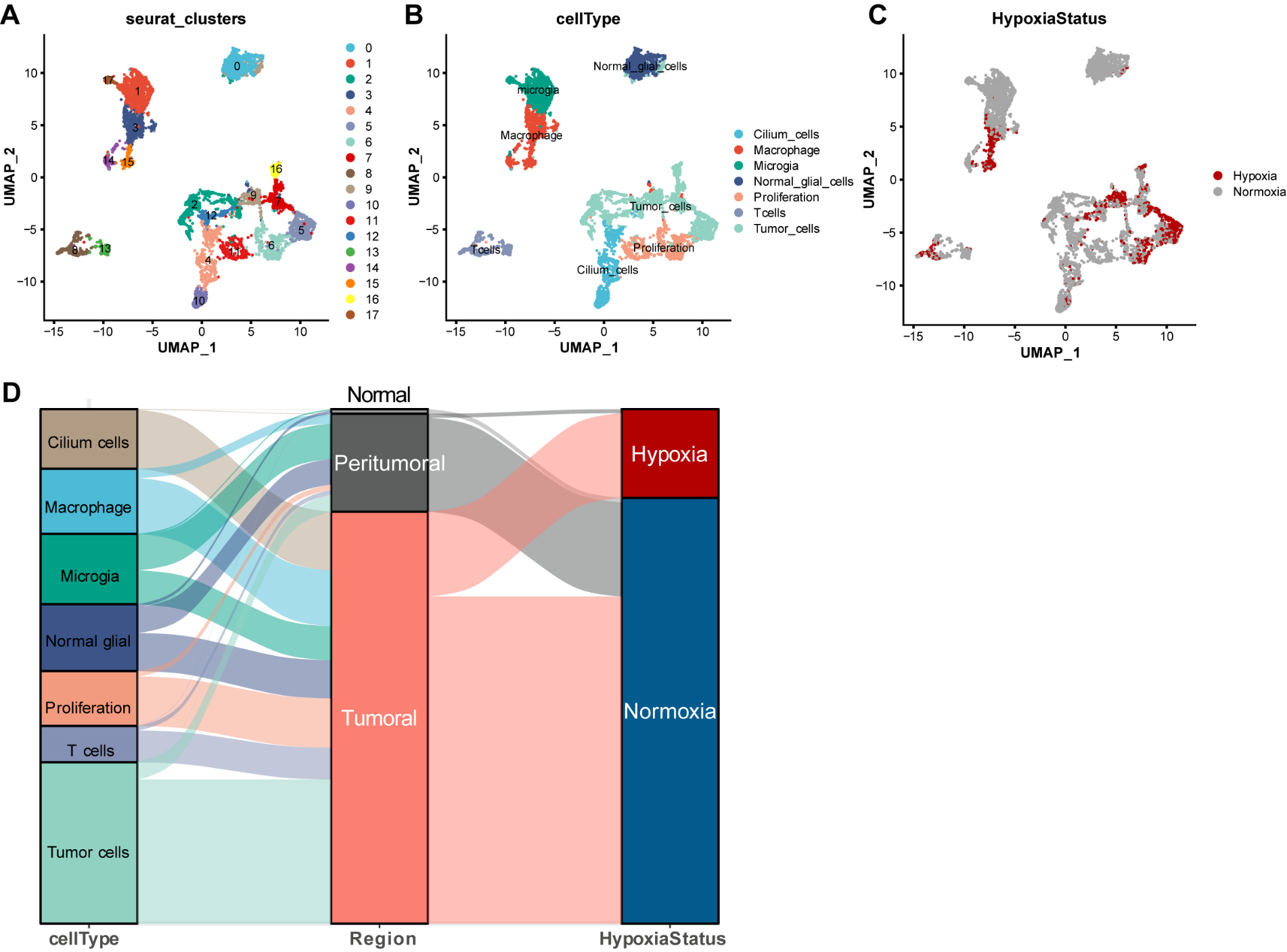


**Supplementary Figure S2 Distribution of hypoxic cells in GSE117891.** (A) UMAP plot of all the 6,148 single cells in GBMs. (B) UMAP plot of all single cells, colored by cell types. (C) UMAP plot of all single cells, colored by hypoxia status. (D) Sankey diagram showed the distribution of hypoxic cells across different cell types and tissue regions.


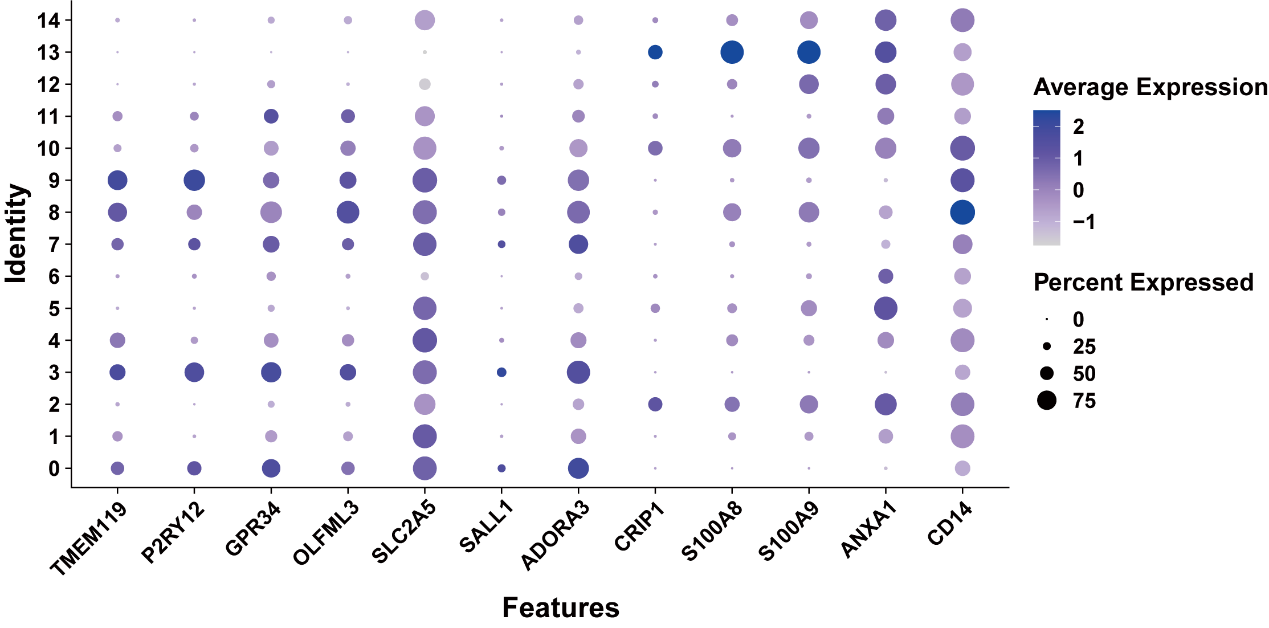


**Supplementary Figure S3 Immune cells re-annotation.** Canonical marker genes expression level of macrophages and microglia (TMEM119, P2RY12, GPR34, OLFML3, SLC2A5, SALL1, ADORA3 for microglia and CRIP1, S100A8, S100A9, ANXA1, and CD14 for macrophages).


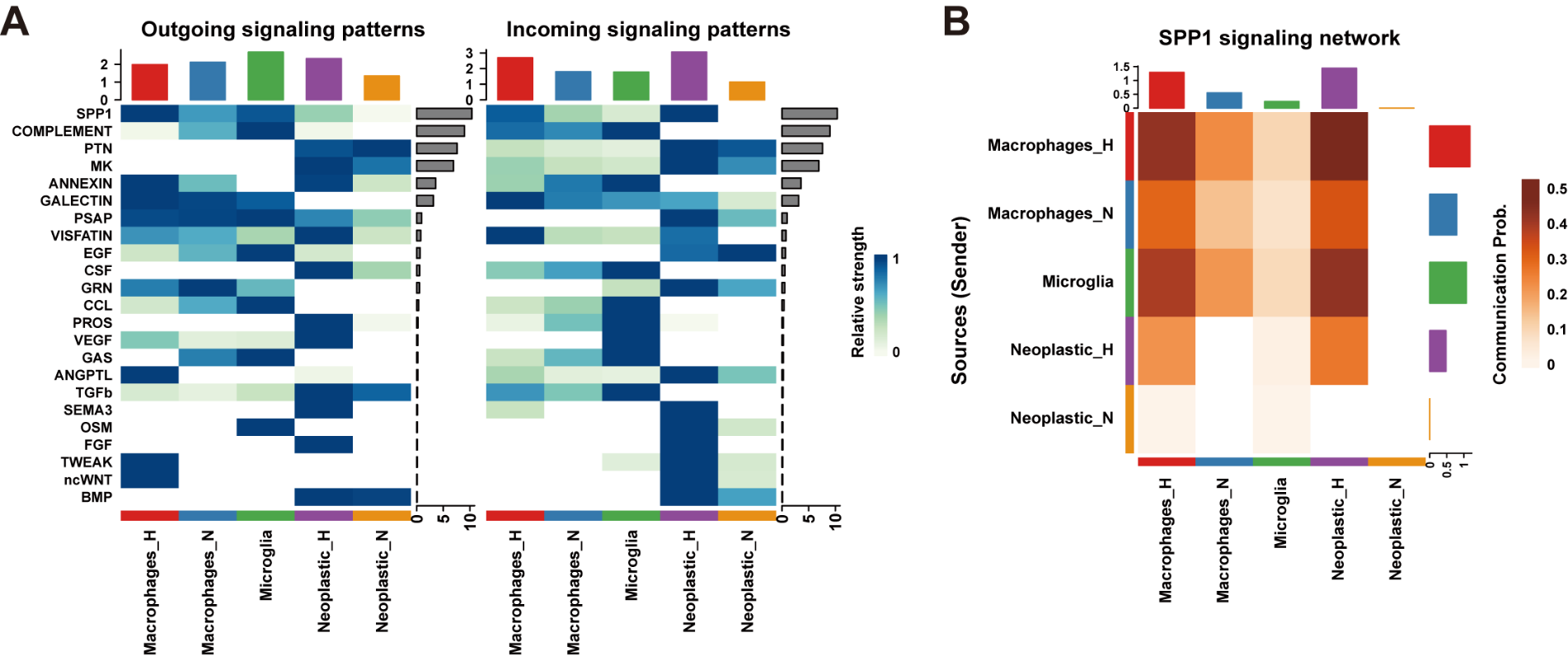


**Supplementary Figure S4 Cell-cell communication between immune cells and tumor cells.** (A) Signals incoming and outgoing patterns. (B) SPP1-related signaling networks. Y axis refers to cells that send the signal, whereas x axis refers to cells that receive the signal.


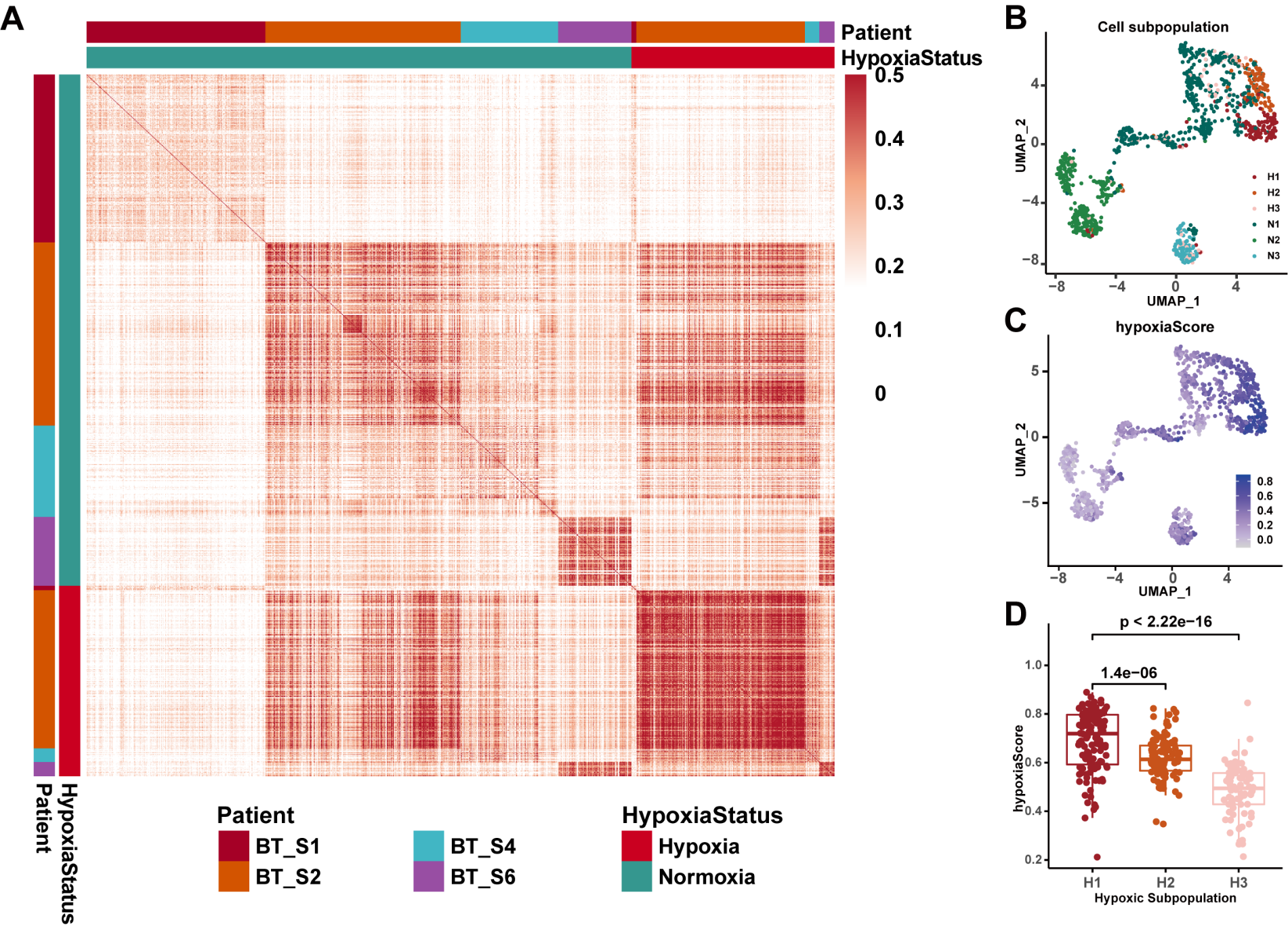


**Supplementary Figure S5 Hypoxia heterogeneity in tumor cells.** (A) Correlation analysis of all the tumor cells in hypoxia status or normoxia status from four patients. (B) UMAP plot of all the tumor cells (colored by subpopulations). (C) UMAP plot of all the tumor cells (colored by hypoxia score). (D) Hypoxia scores of three hypoxic tumor subpopulations. The P value is calculated by the wilcoxon rank sum test.


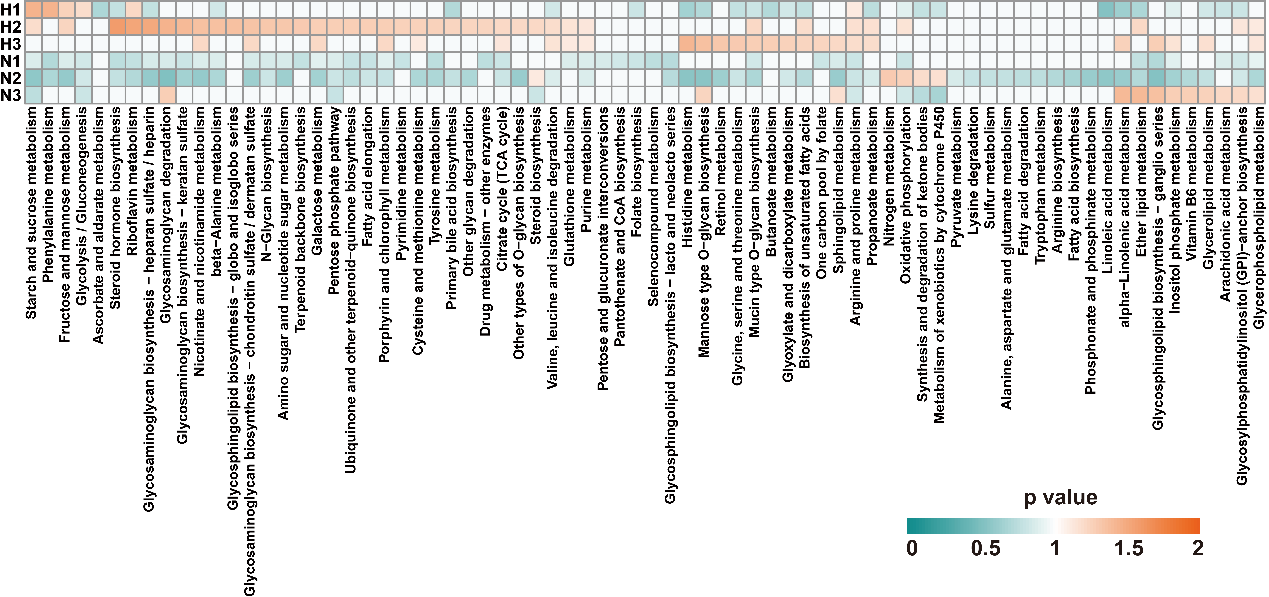


**Supplementary Figure S6 Metabolic pathway activities in each subpopulation.** Statistically non-significant values (random permutation test p > 0.05) were shown as blank.


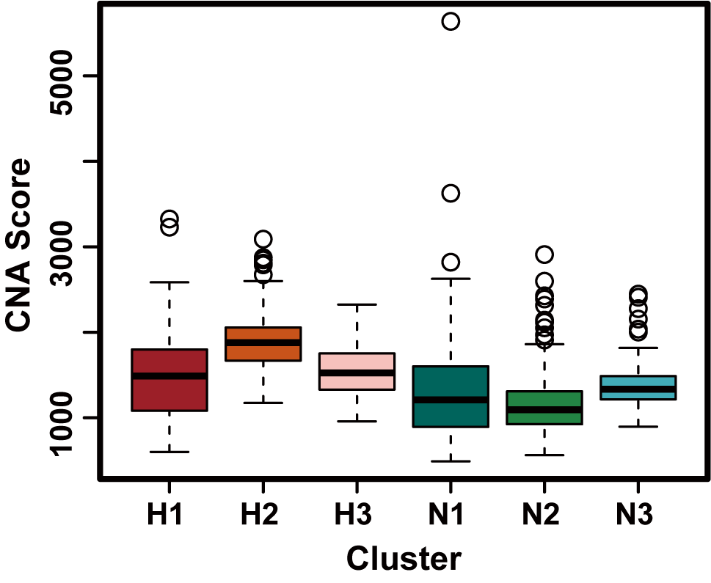


**Supplementary Figure S7 InferCNV analysis of six subpopulations.** CNV scores of each subpopulation.


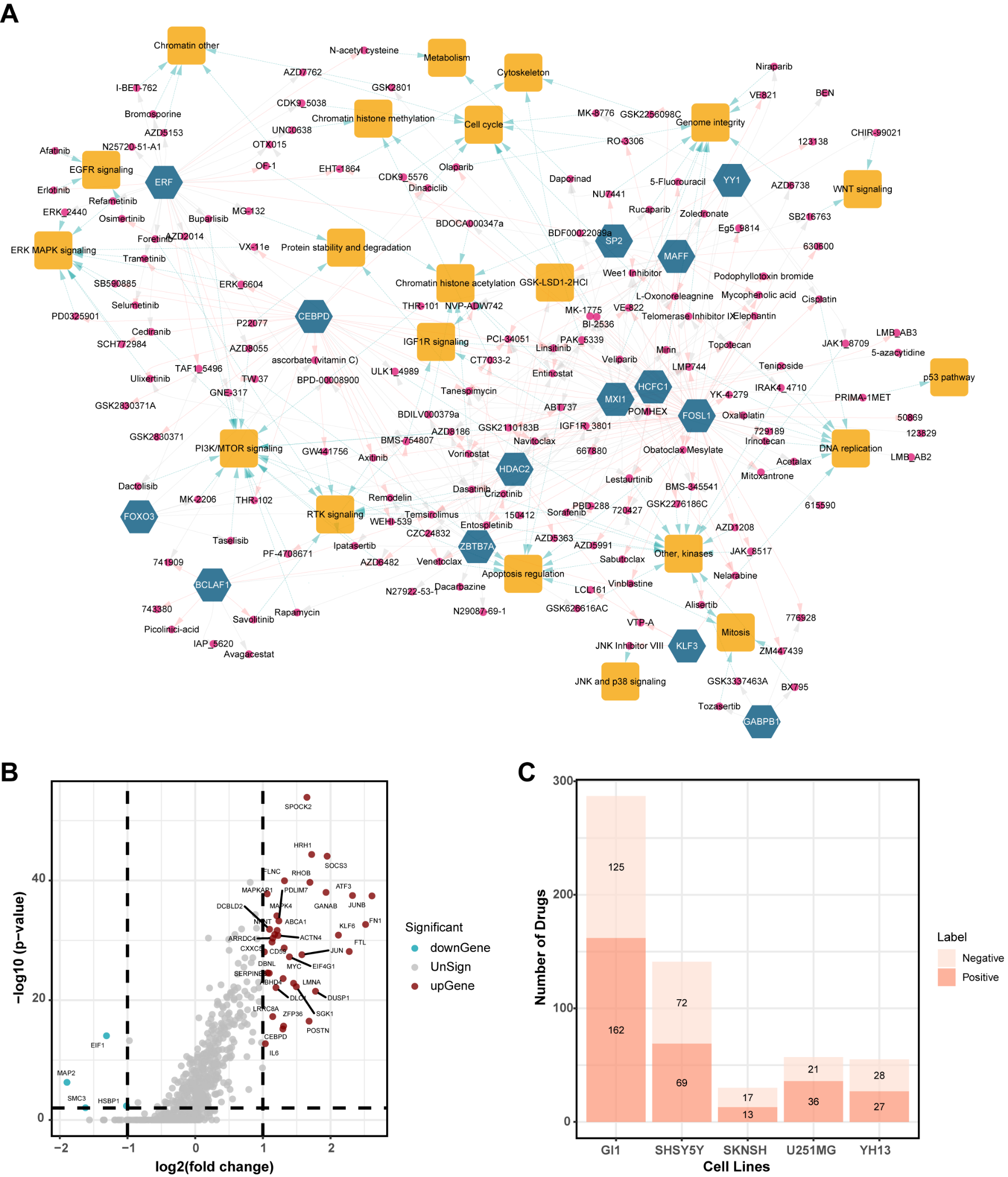


**Supplementary Figure S8 Potential drugs targeting critical regulators.** (A) The network of all critical TFs-related drugs and signaling pathways. The pink line indicated a positive correlation and the gray line indicated a negative correlation. Blue dotted line indicated the relationship between drugs and regulated signaling pathways. (B) Volcano plots of differential expression genes targeted by critical TFs of H2 and N2 subpopulation. Genes selected for differential expression analysis were previously identified regulators and their targeted genes. Genes were labeled if p < 0.05 and |log2(fold change)| > 1. (C) Numbers of candidate drugs perturbed by DEGs of critical TFs targeted in five brain cancer cell lines.

**Supplementary Tables**

Supplementary Table S1. Description of the hypoxia gene signatures.

Supplementary Table S2. Cell type-specific important hypoxia-related gene signatures.

Supplementary Table S3. The performance of the CHPF in each dataset.

Supplementary Table S4. Marker genes of each tumor subpopulation.

Supplementary Table S5. Gene list of WGCNA modules.

Supplementary Table S6. Description of the four cancer-related signatures.

Supplementary Table S7. Univariate Cox proportional hazards regression of critical TFs.

Supplementary Table S8. Description of the brain cancer cell lines in GDSC database.

Supplementary Table S9. Spearman correlation between drugs and critical TFs.

| **Supplementary Table S1. Description of hypoxia gene signatures.** | | |
| --- | --- | --- |
| **Gene Set Name** | **Description** | **PMID** |
| LEONARD_HYPOXIA | Genes up-regulated in HK-2 cells kidney tubular epithelium) under hypoxia and down-regulated on re-oxygenation. | 12885785 |
| HARRIS_HYPOXIA | Genes known to be induced by hypoxia | 11902584 |
| HALLMARK_HYPOXIA | Genes up-regulated in response to low oxygen levels (hypoxia). | 26771021 |
| MENSE_HYPOXIA_UP | Hypoxia response genes up-regulated in both astrocytes and HeLa cell line | 16507782 |
| ELVIDGE_HYPOXIA_UP | Genes up-regulated in MCF7 cells (breast cancer) under hypoxia conditions | 16565084 |
| FARDIN_HYPOXIA | Genes in the hypoxia signature, based on analysis of 11 neuroblastoma cell lines in hypoxia and normal oxygen conditions. | 20624283 |
| KIM_HYPOXIA | Genes up-regulated in normal fibroblasts under hypoxia conditions. | 14499499 |

**Supplementary Table S3. The performance of the CHPF in each dataset.**

| **Datasets** | **GSE117891** | **GSE125587** | **GSE131928** |
| --- | --- | --- | --- |
| Cells Number | 6148 | 21750 | 16201 |
| High-confidence hypoxic Cells | 213 | 7028 | 1062 |
| High-confidence normoxic cells | 3112 | 4912 | 3765 |
| Accuracy | 0.991 | 0.9345 | 0.9925 |
| 95%CI | (0.9835, 0.9957) | (0.928, 0.9406) | (0.9882, 0.9956) |
| Kappa | 0.9311 | 0.8665 | 0.9785 |
| Sensitivity | 1.00000 | 0.9078 | 0.9851 |
| Specificity | 0.99034 | 0.9736 | 0.9947 |
| Precision | 0.87952 | 0.9805 | 0.9815 |
| Recall | 1.00000 | 0.9078 | 0.9851 |
| F1 | 0.93590 | 0.9428 | 0.9833 |

| **Supplementary Table S6. Description of the four cancer-related signatures.** | | |
| --- | --- | --- |
| **Gene Set Name** | **Description** | **PMID** |
| Apoptosis | The inactivation of apoptosis in cancer cells lead to the persistence of such grossly abnormal cells in the tissues. | 30329142 |
| Angiogenesis | Angiogenesis ensures that cancer cells receive continuous supplies of oxygen and other nutrients. | 30329142 |
| EMT | EMT has been indicated to be involved in the initiation of metastasis in cancer progression and in acquiring drug resistance. | 30329142 |
| invasion | Invasion is a critical carcinogenic event in which cancer cells escape from their primary sites and spread to blood or lymphatic vessels. | 30329142 |

**Supplementary Table S7. Univariate Cox proportional hazards regression of critical TFs.**

| **TFs** | **coefficient** | **HR (95% CI)** | **P-value** |
| --- | --- | --- | --- |
| MXI1 | -0.2059 | 0.8139(0.7211-0.9186) | 0.00085 |
| CEBPD | 0.1899 | 1.2091(1.0741-1.3611) | 0.00167 |
| FOSL1 | 0.1389 | 1.149(1.0414-1.2677) | 0.00563 |
| MAFF | 0.117 | 1.1241(1.0296-1.2273) | 0.00906 |
| HDAC2 | -0.1429 | 0.8668(0.7655-0.9816) | 0.02432 |
| YY1 | -0.2813 | 0.7548(0.5845-0.9747) | 0.03107 |
| BCLAF1 | -0.0651 | 0.937(0.7975-1.1007) | 0.42821 |
| HCFC1 | -0.1026 | 0.9025(0.6766-1.2038) | 0.48512 |
| KLF3 | 0.0657 | 1.0679(0.8701-1.3107) | 0.52945 |
| FOXO3 | -0.0377 | 0.963(0.8367-1.1084) | 0.5992 |
| ZBTB7A | 0.1084 | 1.1145(0.6617-1.8771) | 0.68365 |
| ERF | 0.0181 | 1.0183(0.8753-1.1846) | 0.81458 |
| SP2 | -0.0362 | 0.9645(0.6608-1.4076) | 0.85117 |
| GABPB2 | -0.0128 | 0.9873(0.8443-1.1544) | 0.87233 |
